# Genetic investigation of dementias in clinical practice

**DOI:** 10.1590/0004-282X-ANP-2022-S103

**Published:** 2022-08-12

**Authors:** Leonel Tadao Takada

**Affiliations:** 1, Faculdade de Medicina, Hospital das Clinicas, Divisão de Clínica Neurológica, São Paulo SP, Brazil.

**Keywords:** Alzheimer Disease, Frontotemporal Dementia, Genetic Testing, Dementia, Vascular, Leukoencephalopathies, Doença de Alzheimer, Demência Frontotemporal, Testes Genéticos, Demência Vascular, Leucoencefalopatias

## Abstract

**Background:**

The field of neurodegenerative dementia genetics has advanced significantly over the past two decades, but there are still more to be discovered (such as the gene mutation in some familial forms of dementia).

**Objective:**

to provide a brief review of the most recent discoveries regarding monogenic dementia, and covering the most frequent genetic diseases that can cause dementia (neurodegenerative or not).

**Methods:**

a review of the literature will be carried out.

**Results:**

neurodegenerative dementias, vascular dementias and leukoencephalopathies caused by single pathogenic variants are presented.

**Conclusion:**

The spectrum of clinical presentations for most of the genes discussed is wide, and hence genetic testing in clinic should try to cover as many genes as possible.

## RESUMO

**Antecedentes:**

O campo da genética das demências neurodegenerativas avançou significativamente nas últimas duas décadas, mas ainda há mais a ser descoberto (como a mutação genética em algumas formas familiares de demência).

**Objetivo:**

fornecer uma breve revisão das descobertas mais recentes sobre demência monogênica, e abrangendo as doenças genéticas mais frequentes que podem causar demência (neurodegenerativa ou não)**. Métodos:** será realizada uma revisão da literatura.

**Resultados:**

são apresentadas demências neurodegenerativas, demências vasculares e leucoencefalopatias causadas por variantes patogênicas únicas.

**Conclusão:**

O espectro de apresentações clínicas para a maioria dos genes discutidos é amplo e, portanto, os testes genéticos na clínica devem tentar cobrir o maior número possível de genes.

## INTRODUCTION

The genetics of neurodegenerative dementias is a fast-growing field of research. Now many monogenic causes of dementia have been identified. Polygenic or complex forms of dementia are better understood now because of genetic studies, such as genome-wide association studies - GWAS, as new proteins and pathophysiological pathways have been found. But there s still much to be done, as many families still await the identification of their gene mutation. 

It is in this scenario that we stand. When we see patients with familial forms of dementia, we must decide whether that patient should undergo genetic testing or not. Before the testing is ordered, we should be able to counsel families and patients regarding the pros and cons of moving forward. We must think of the patient´s children, who are potentially at risk of bearing a disease-causing mutation and know what and how to speak to them. We must accept the fact that even with exome sequencing, the gene might not be identified (and this is not a rare occurrence). Or that a variant of unknown significance might be found (and what we can do if that occurs). Or that a pathogenic variant can be found in a non-related gene (such as a pathogenic variant in BRCA1). 

In this paper, we will try to summarize what is currently known about monogenic forms of dementia and present the main considerations that we should be aware of when genetic testing is an alternative to identify a patient's disease. 

## ALZHEIMER'S DISEASE

Neurodegenerative diseases have been defined by the main altered protein that is found in inclusions. In the case of Alzheimer´s disease (AD), for example, beta amyloid and tau are found in amyloid plaques and neurofibrillary tangles, respectively, and those two proteins are necessary for a definite diagnosis of AD. Clinically, AD most frequently present as a dementia of the Alzheimer type (DAT), in which episodic memory is the most and the earliest cognitive domain impaired^1^. But there are clinically atypical forms of AD, such as the logopenic variant of primary progressive aphasia (PPA), posterior cortical atrophy (PCA), the frontal variant of AD, and corticobasal syndrome (CBS)^2^. Therefore, the same proteinopathy might have different clinical presentations and it is relevant to genetic testing, as the genes with pathogenic variants will inevitably be associated with certain proteinopathies. For example, mutations in the presenilin 1 (*PSEN1*), presenilin 2 (*PSEN2*), and *APP* (amyloid precursor protein) genes lead to the accumulation of amyloid beta and tau^1^, without necessarily leading to a DAT phenotype. From this we can conclude that it is the areas and networks most affected that will define the clinical presentation, not the abnormal proteins^3^. 

The mean age at onset is around 44 years in *PSEN1* pathogenic variants carriers and ranges from the second to the eighth decades of life. In the case of *APP* pathogenic variant carriers, mean age at onset occurs at around 49 years and the range is from the third to the eighth decades of life. The APP gene is located at chromosome 21, which is the reason why individuals with Down's syndrome tend to develop AD more frequently and at an earlier age. The mean age at onset of the rare variants in *PSEN2* mutations occurs a bit later, at around 55 years of age. The range is also wide, from the fourth to the ninth decades of life^4^ ^,^ ^5^. Monogenic AD is essentially early onset AD, as mutations in those three genes rarely cause disease that begins after 65 years of age.

*PSEN1* pathogenic variant carriers have been reported (tough much less frequently than early onset DAT) to also present as behavioral variant frontotemporal dementia (bvFTD), Parkinson´s disease, spastic paraparesis, corticobasal syndrome, dementia with Lewy bodies (DLB), non-fluent variant PPA, and cerebral amyloid angiopathy. *PSEN2* mutations have been found in cases of Parkinson's disease, bvFTD, semantic variant PPA, DLB, and nfvPPA. APP pathogenic variants or duplication of the gene have been associated with Parkinson's disease dementia, nfvPPA, cerebral amyloid angiopathy, hereditary cerebral hemorrhage with amyloidosis, and vascular dementia^4^.

## FRONTOTEMPORAL LOBAR DEGENERATION

Another group of neurodegenerative diseases that is relevant to genetic testing is frontotemporal lobar degeneration (FTLD). The abnormal proteins are tau (in around 45% of cases), TDP-43 (in around 50% of cases) and the FET family of proteins (of which FUS is the most well-known) in 5-10% of cases^6^. Clinically, FTLD most frequently present as bvFTD, nfvAPP, svAPP, progressive supranuclear palsy (PSP) or CBS (we call this group of phenotypes frontotemporal dementia - FTD)^6^. Around 15% of patients with FTD develop motor neuron disease (most frequently amyotrophic lateral sclerosis - ALS). 

The field of FTD genetics has been growing steadily over the past decade. The first FTLD-associated gene was the microtubule associated protein tau gene (*MAPT*), in 1998. In 2006, another breakthrough came with the discovery of the progranulin (*GRN*) gene. In 2011, the third “big” (in terms of frequency) gene was identified. Hexanucleotide repeat expansions in *C9ORF72* were discovered as the most frequent genetic cause of FTD-ALS^7^. Now there are more than 20 genes associated with FTLD. Pathogenic variants in *MAPT* most frequently cause bvFTD (45%), Parkinson's disease (5%), PSP (4%) and one third of cases have dementia without a clear phenotype^8^. Mean age at onset of symptoms is 50 years and the range is 17 to 82 years. Pathogenic variants in *GRN* have a mean onset of symptoms at age 61 years, ranging from 25 to 90 years. Thirty-eight percent of patients develop bvFTD, 9% nfvPPA, 8% DAT, 4% CBS, 3% PPA (not specified) and here also a third of the patients have dementia not otherwise specified^8^. 

Many of the genes cause FTD and/or ALS, such as the TDP-43 gene (*TARDBP*) or the *FUS* gene. Most of those genes are rare causes of FTD and explain a small amount of familial ALS cases. But *C9ORF72* is the most common monogenic cause of FTD-ALS. In *C9ORF72*-associated disease, mean age at onset occurs at 58 years (range 26-97). The most common phenotypes are bvFTD (31%), ALS (19% in a dementia predominant cohort), and FTD-ALS (11%). Six percent of patients receive a diagnosis of DAT and 25% have dementia not otherwise specified^8^. 

There is a group of genes in which pathogenic variants cause a complex syndrome called multisystem proteinopathy (MSP)^9^. The main gene in this group is the valosin-containing protein gene (*VCP*), but others have been described such as the *SQSTM1* (Sequestosome 1) gene. The MSP phenotype is a combination of Paget's disease of the bone, myopathy (particularly inclusion body myopathy), FTD (mainly bvFTD) and, less frequently, ALS. The clinical presentation (even within families) is highly heterogeneous, and each person of a family may present with a certain phenotype or a combination of phenotypes. In the case of *VCP* mutations, FTD has a penetrance of 30%^9^. 

Regarding gene-proteinopathy correlations, mutations in *MAPT* cause tau pathology, mutations in the *FUS* gene cause FUS pathology and in the other genes cause TDP-43 pathology^7^. 

## DEMENTIA WITH LEWY BODIES

Familial DLB appears to be rare. In a review published in 2017, Vergouw and cols. reported 13 patients with monogenic DLB. Four of those cases were due to mutations in the α-synuclein gene (*SNCA*), one due to the p.G2019S variant in *LRRK2*, seven due to mutations in one of the AD-causing genes, and one with a pathogenic variant in the β-synuclein gene (*SCNB*). Pathogenic variants in *SNCA* are a cause of monogenic Parkinson's disease (PD) and more than half of the patients develop cognitive decline. Only two of those cases had another DLB case in the family; the rest had either PD/PD dementia or AD family history^10^. 

## VASCULAR DEMENTIA

### Cadasil

Cerebral autosomal dominant arteriopathy with subcortical infarcts and leukoencephalopathy (CADASIL) is an autosomal dominant, not fully penetrant disorder caused by mutations in the *NOTCH3* (Notch Receptor 3) gene^11^. 

Patients start to develop migraines with aura and depression in early adulthood. Subcortical or lacunar strokes start to appear at the fifth decade of life, followed by cognitive decline and death by the sixth decade of life^12^ ^,^ ^13^. 

Brain MRI changes appear 10-15 years before the onset of symptoms. They begin as multifocal brain changes, but with the progression of disease, also involve the external capsule and the temporal poles, as well as periventricular and frontoparietal regions. T2* sequences also reveal small foci of micro hemorrhages.

Other rare forms of monogenic vascular dementia include the recessive disorder cerebral autosomal recessive cerebral arteriopathy with subcortical infarcts and leukoencephalopathy (CARASIL), caused by mutations in *HTRA1* (HtrA Serine Peptidase 1). More recently, the autosomal dominant cathepsin A-related arteriopathy with strokes and leukoencephalopathy (CARASAL) caused by pathogenic variants in *CTSA* (Cathepsin A) was described^12^.

### Prion diseases

Around 15% of prion diseases are caused by mutations in the prion protein gene (*PRNP*). The pathogenic variants in *PRNP* might be missense (the most common), octapeptide repeat insertions, or nonsense^14^. 

Missense variants can cause genetic Creutzfeldt-Jakob disease (gCJD), Gerstman-Sträussler-Scheinker syndrome (GSS) or Fatal Familial Insomnia (FFI). gCJD presents as rapidly progressive dementia, with visual, cerebellar, pyramidal and/or extrapyramidal features. Ancillary testing findings (such as cortical ribboning in MRI DWI) are not as accurate in gCJD as they are in sporadic CJD. GSS is typically slowly a progressive ataxia with late cognitive decline. Patients with FFI develop progressive incurable insomnia, with later visual hallucinations, dysautonomia, and cognitive decline. Death occurs in months. Octapeptide repeat insertions tend to present as gCJD if the number of repeats is six or less, and present as GSS if the number of repeats is six or more. The most common missense mutation worldwide is E200K. This variant is associated with the gCJD phenotype and is clinically indistinguishable from sporadic CJD. Of note, 50% of gCJD due to E200K do not have a positive family history. This is why experts in the field recommend that in all cases of CJD, even if it appears to be sporadic, testing for *PRNP* pathogenic variants is recommended. 

Another interesting aspect of the genetics of prion disease is that the pathogenic variant associated with FFI is D178N with methionine in codon 129 in the same allele (cis); whereas if the cis codon 129 is valine, the phenotype is gCJD^14^. 

## LEUKOENCEPHALOPATHIES / DYSTROPHIES

Leukodystrophies are disorders that affect predominantly the white matter (glial cells and myelin sheath) of the nervous system and are determined by pathogenic variants in certain genes. Leukodystrophies are more common in children, but over time leukodystrophies in adulthood have been discovered and characterized. Here we will comment on the most frequent and relevant types of adulthood leukodystrophies. 

The diagnosis of leukodystrophies is often a challenge. Sometimes the clinical presentation is not specific, the disease might be sporadic, and the mutation might be a repeat expansion that is not detected by current whole exome or genome sequencing methods. It should be noted that even though we have advanced significantly in the genetic diagnosis of leukodystrophies, in a study from 2017, more than 70% of cases did not receive a genetic diagnosis after whole exome sequencing^12^.

More than 90 leukodystrophies and genetic leukoencephalopathies have been characterized to date^15^. I will comment on a few leukoencephalopathies with adult onset and cognitive decline. 

## X-LINKED ADRENOLEUKODYSTROPHY

 This leukodystrophy is caused by mutations in the *ABCD1* (ATP Binding Cassette Subfamily D Member 1) gene. It has two clinical phenotypes: adrenomyeloneuropathy with ages at onset of symptoms ranging from 14 to 60 years (typically 20-30 years)^15^, and which presents with spastic paraparesis, bladder and sexual dysfunction. Adrenal insufficiency can be observed in these patients. The other phenotype is called adulthood cerebral adrenoleukodystrophy and typically begins to affect male individuals 21 years or older. Early behavioral changes and psychosis with late spastic paraparesis, ataxia, seizures and dementia are the most often seen symptoms. Very long chain fatty acids are increased in the serum. Most of the patients are male, but there have been reports of females developing symptoms^12^. 

 In the brain MRI, both parieto-occipital and frontal T2/FLAIR hyperintensities can be found, as well as hyperintensities in the splenium of the corpus callosum. The combination of both patterns is associated with a more severe disease. Contrast enhancement might be observed.

## METACHROMATIC LEUKODYSTROPHY

 Metachromatic leukodystrophy is an autosomal recessive disorder caused by mutations in the arylsulfatase gene (*ARSA*) or rarely in the prosaposin (*PSAP*) gene. There are late infantile, juvenile and adult presentations. The adult form is characterized by cognitive impairment, behavioral changes, and occasionally psychosis, or peripheral neuropathy^15^. Brain MRI shows symmetric periventricular white matter T2/FLAIR hyperintensities and cortical atrophy^12^. Diagnosis is suspected by low arylsulfatase A levels, and confirmed by genetic sequencing. 

## CEREBROTENDINEOUS XANTHOMATOSIS

 This is an autosomal recessive disease caused by pathogenic variants in the *CYP27A1* (Cytochrome P450 Family 27 Subfamily A Member 1) gene^12^. Patients develop xanthomas in the Achilles and other tendons, during adolescence or adulthood. The adult form of the disease is mainly neurological, with cognitive decline, ataxia, spasticity, and neuropsychiatric symptoms. In the brain MRI, the cerebellum is particularly affected, with T1 hypointense and T2 hyperintense lesions in the dentate nuclei. Hyperintensities in the white matter of the cerebellum can also be observed. The diagnosis of cerebrotendineous xanthomatosis is confirmed by high serum cholesterol and the treatment (which should be instituted early for better outcomes) is chenodeoxycholic acid or cholic acid^15^. 

## HEREDITARY DIFFUSE LEUKODYSTROPHY WITH AXONAL SPHEROIDS AND PIGMENTED GLIA

Onset of symptoms occur between 40-70 years, and the most significant symptoms are cognitive and neuropsychiatric (the dementia phenotype might resemble that of bvFTD). Gait changes can occur, as well as seizures and bladder dysfunction. This is an autosomal dominant disorder, but the penetrance is incomplete and cases with *de novo* mutations have been reported in the literature. This leukodystrophy is caused by mutations in the *CSF1R* (Colony Stimulating Factor 1 Receptor) gene. Brain MRI white matter T2/FLAIR hyperintensities are predominant in the frontal regions (early in the disease course, restricted diffusion might be found). SWI hypointensities indicating calcifications in frontal and parietal regions can also be found^12^ ^,^ ^15^.

## KRABBE DISEASE

 This is a rare autosomal recessive disease caused by variants in the *GALC* (Galactosylceramidase) gene. There are infantile, juvenile and adult forms of the disease. The adult form is usually mild and typically presents, with spasticity, peripheral neuropathy, dementia, ataxia, and visual loss. Corticospinal tracts and optic radiations as well as T2/FLAIR hyperintensities are the abnormalities reported in Krabbe disease patients^12^. Confluent periventricular or parieto-occipital T2 hyperintensities can also be reported in patients^15^.

## POLYCYSTIC LIPOMEMBRANOUS OSTEODYSPLASIA WITH SCLEROSING LEUKOENCEPHALOPATHY (NASU HAKOLA DISEASE)

Homozygous pathogenic variants in *TREM2* (Triggering Receptor Expressed On Myeloid Cells 2) and *TYROBP* (TYRO protein tyrosine kinase-binding protein) can cause a syndrome characterized by bone pain, cysts, and fractures that begin to appear in the third decade of life, and a frontal syndrome (similar to bvFTD) that typically occurs in the fourth decade. Death occurs at around 50 years. Bone radiography shows fractures and cysts, predominantly in the extremities. Neuroimaging is characterized by basal ganglia calcifications and periventricular T2-weighted hyperintensities, particularly in the frontal lobes^16^. Families with the bvFTD phenotype only (without bone involvement) have also been reported in the literature.

## GENETIC TESTING IN CLINICAL PRACTICE

The first step to do when seeing a patient with dementia is to build a family pedigree with three generations. By doing that, we are able to identify if the patient has no relevant family history, if the patient has family history, but it is only of late onset AD (and in this case we tend not to pursue genetic testing - around 20% of late onset AD cases have a positive family history but they are mostly not autosomal dominant), or if the patient has a family history composed of only one individual with the same diagnosis or if the family history is suggestive of an autosomal dominant pattern of inheritance. Virtually all of the neurodegenerative dementias that are monogenic are autosomal dominant with high penetrance. As previously mentioned, some of the leukodystrophies are recessive and penetrance might be lower and hence those possibilities should also be kept in mind. 

If a patient has early onset AD and one or two first degree relatives with early onset AD, this is a patient to whom genetic testing should be considered. Same thing for a patient who has bvFTD and had a mother diagnosed with early onset AD (it was not uncommon in the past for patients to be misdiagnosed and a patient with bvFTD be diagnosed as AD) and a sister with nfvPPA. If a genetic test is ordered, it is fundamental to be sure that the patient and his/her family understands the consequences of finding a pathogenic variant, as well as the possibility of finding a novel variant of unknown significance, or not finding anything. Also, it is important to decide which test will be ordered. Nowadays, Sanger sequencing of each gene at a time is only reserved to confirm variants found in next generation sequencing (NGS). We can order panels of genes (related to dementias, with 40-60 genes), order a whole exome sequencing, or whole genome sequencing. Whole genome sequencing is not always available for clinical purposes. Requesting a panel will allow those specific genes to be sequenced with a greater depth than whole exome sequencing (WES) but of course the number of genes tested is much lower. The cost is different, but the difference has decreased over the years so that ordering a WES ends up being - in most cases - the most cost-effective choice. It should be noted that long repeat expansions such as the one found in *C9ORF72* are not detected by current NGS methods, and thus must be tested separately. 

In our previously unpublished experience with autosomal dominant AD, of 18 probands from different families, only eight (44%) had pathogenic variants in *PSEN1* (n=6) or *APP* (n=2). And among those eight, two had variants of unknown significance (which we were later able to prove their pathogenicity; Takada et al., submitted). And regarding FTD (also unpublished), of 19 probands, 32% had pathogenic variants in progranulin, 11% had *C9ORF72* repeat expansions, and 10% had *MAPT* mutations. This means more than 40% did not have mutations in the three major FTD genes. In a study^17^, WES combined with the identification of *C9ORF72* repeat expansions could identify a monogenic cause in 14 of 41 (34%) familial cases of FTD. 

Once a pathogenic variant is identified in the proband, his or her children are put at 50% each risk of having the same variant. Some of the children might not want to pursue predictive testing, but some want (for different reasons, such as the possibility of doing a preimplantation genetic diagnosis to exclude the possibility that their offspring have the same variant, or to plan their lives^18^). It is essential to perform genetic counseling to those individuals who are considering predictive testing. They must be assessed by a neurologist and a psychiatrist prior and after testing^19^. Some individuals are simply not prepared to receive presymptomatic testing results, and those should be firmly recommended not to be tested at that time.

If a variant of unknown significance is found in a gene that is relevant to the phenotype of the proband, one thing that can be done is a segregation study. If we can show that the variant runs in the family together with the symptomatic cases (and that older asymptomatic individuals do not carry the variant), this is evidence of pathogenicity (though not conclusive evidence). 

In conclusion, we are possibly currently seeing the development of treatments for some of the genetic forms of disease that were presented here. The DIAN (dominantly inherited Alzheimer network) study is currently recruiting to a study that will use an anti-tau (E2814) and an anti-amyloid (lecanemab) drugs in combination. The group at Antioquia, Colombia is currently studying an anti-amyloid drug called crenezumab to treat individuals with a mutation in the *PSEN1* gene. 

*GRN* pathogenic variants cause disease by haploinsufficiency, which means the allele with the variant does not produce enough progranulin to maintain normalcy. There are currently at least four drugs being tested for GRN-associated FTD, which is very exciting. The prospect of finding disease-modifying drugs in the near future for monogenic forms of neurodegenerative dementia is real, though many other forms associated with other genes are still waiting for their clinical trials. 
